# Among Persons With Multiple Sclerosis (MS), Objective Sleep, Psychological Functioning, and Higher Physical Activity Scores Remained Stable Over 2 Years—Results From a Small Study Under Naturalistic Conditions

**DOI:** 10.3389/fpsyt.2020.586244

**Published:** 2020-12-14

**Authors:** Dena Sadeghi Bahmani, Roman Gonzenbach, Jürg Kesselring, Jens Bansi, Robert W. Motl, Dominik Cordier, Oliver Rothen, Daryl Niedermoser, Markus Gerber, Serge Brand

**Affiliations:** ^1^Center of Affective, Stress and Sleep Disorders (ZASS), Psychiatric Clinics (UPK), University of Basel, Basel, Switzerland; ^2^Departments of Physical Therapy, University of Alabama at Birmingham, Birmingham, AL, United States; ^3^Sleep Disorders Research Center, Kermanshah University of Medical Sciences (KUMS), Kermanshah, Iran; ^4^Kliniken Valens, Valens, Switzerland; ^5^Department of Neurosurgery, University Hospital, University of Basel, Basel, Switzerland; ^6^Division of Sport Science and Psychosocial Health, Department of Sport, Exercise and Health, Faculty of Medicine, University of Basel, Basel, Switzerland; ^7^Substance Abuse Prevention Research Center, Health Institute, Kermanshah University of Medical Sciences (KUMS), Kermanshah, Iran; ^8^School of Medicine, Tehran University of Medical Sciences, Tehran, Iran

**Keywords:** multiple sclerosis, sleep, electroencephalography, physical activity, naturalistic conditions, stability

## Abstract

**Background:** Persons with multiple sclerosis (PwMS) are at increased risk to report poor sleep patterns and lower physical activity indices. To date, data on longitudinal objectively sleep assessment is missing. In the present study, we investigated the pattern of objective sleep and subjective physical activity indices over a period of 13.5 months, under naturalistic conditions.

**Method:** 13.5 months after their first assessment, a total of 16 PwMS (mean age = 49.13 median EDSS score: 5; 11 females) were reassessed on their objective sleep via portable sleep-electroencephalogram (EEG-) devices, along with their subjective sleep patterns (symptoms of insomnia, restless legs syndrome (RLS), and sleep-disordered breathing), physical activity indices, psychological functioning (symptoms of depression, fatigue, daytime sleepiness), and MS-related information (fatigue, EDSS; disease-modifying treatments). While the baseline assessment was performed in a rehabilitation center, the follow-up assessment took place at participants' naturalistic and familiar setting.

**Results:** Statistically, symptoms of depression and fatigue, subjective sleep, and physical activity levels did neither increase, nor decrease over time, although descriptively, both moderate and vigorous physical activity levels decreased, and fatigue and subjective insomnia increased. Time awake after sleep onset statistically significantly decreased, while light sleep duration increased by trend.

**Conclusions:** Among a smaller sample of PwMS, objective sleep in their naturalistic setting remained fairly stable over a mean time lapse of 13.5 months after clinic discharge. Physical activity levels descriptively decreased. The present results are of clinical and practical importance for treatment counseling: PwMS can be reassured that their sleep quality does not deteriorate, once they have left a rehabilitation center. Further, they should be encouraged to keeping their physical activity levels as stable as possible.

## Introduction

Multiple sclerosis is a chronic inflammatory autoimmune disease of the central nervous system (CNS) (1, 2). The immune system is activated against the self and damages myelin layers of axons of the CNS. This damage and its location may result in symptoms such as impaired vision and motor functions, sensory loss, and fatigue, impaired sleep, and cognitive decline. Often, the onset of disease is between 18 and 49 years, though early (before 18 years) and late onset (after 49 years) are also observed (3).

Several reviews (4–7) compared sleep patterns between persons with multiple sclerosis (PwMS) and healthy controls. These reviews reported that compared to healthy controls, PwMS had impaired sleep such as symptoms of insomnia, restless legs syndrome, and sleep-disordered breathing, along with increased daytime sleepiness and fatigue.

Restoring sleep is important for daytime functioning such as cognitive processing (8–11) or emotion regulation (12–18). This holds also true for PwMS. However, surprisingly, longitudinal data on sleep in PwMS are scarce, and to our knowledge, longitudinal data on objective sleep measurements in PmMS are mainly missing. These observations hold also true as regards the associations between sleep patterns and physical activity behaviors. For non-clinical samples (19–28), clinical samples (29, 30), and samples with PwMS (31–36), favorable sleep indices were associated with higher physical activity indices. Despite this, longitudinal studies on the development of sleep and physical activity indices over time are scarce. In a first study (37), we showed that about 2 years after disease onset, patterns of subjective sleep remained basically unchanged; that is to say, PwMS reporting restoring sleep at disease onset also reported unchanged restoring sleep 2 years later. As regards physical activity patterns, over time, both sedentary lifestyle and vigorous physical activity indices decreased, while moderate physical activity indices significantly increased. In a further study, objective sleep was assessed under naturalistic conditions in PwMS's familiar home setting (32): Both objective and subjective sleep dimensions were associated with higher moderate physical activity indices.

Data from interventional studies showed that physical activity improved depression, fatigue, and paresthesia (33, 38). Likewise, higher physical activity levels were associated with favorable objective and subjective sleep dimensions (32), higher emotion regulation and empathy (35), and improved sexual behavior (at least among female PwMS) (39).

With the present study, we aimed at expanding previous research on the following dimensions: First, we investigated, if and if so, to what extent objectively assessed sleep at baseline was associated with objectively assessed sleep on average 13.5 months later at follow-up and assessed under naturalistic conditions in PwMS's home setting. Second, we investigated to what extent physical activity indices remained stable or altered from baseline to follow-up. Third, we explored if and to what extent both objective sleep indices (at baseline and follow-up) and physical activity patterns (at baseline and follow-up) were related to each other. To do so, we combined findings from two previous studies and publications (32, 33). While objective sleep and subjective physical activity indices were main outcome dimensions, symptoms of depression and fatigue and EDSS scores were secondary outcome variables.

The following two hypotheses were formulated. First, based on previous findings (37), we assumed that also objective sleep patterns remained stable over a time lapse of on average 13.5 months. Second, following previous results (37, 40–42), we assumed that physical activity indices remained stable over a time lapse of on average 13.5 months.

## Method

### Study Design

Before their discharge, 46 PwMS treated for their disease at the Kliniken Valens (Valens SG; Switzerland) were thoroughly assessed regarding their objective and subjective sleep, psychological functioning, and illness-related information (33). This timepoint is termed baseline, and all assessments at baseline were performed in the rehabilitation center of the Kliniken Valens. Of those 46 PwMS, 26 had also sleep-EEG assessments. These 26 PwMS were contacted again, and 16 agreed to participate some months later (32); this timepoint is termed follow-up, and all assessments were performed at participants' naturalistic and familiar setting. All participants were fully informed about the aims of the study and the confidential data handling. Thereafter, they signed the written informed consent. Participants completed a booklet of questionnaires covering sleep- and MS-related information. Further, their objective sleep was assessed with portable EEG devices in their naturalistic and familiar setting. As mentioned elsewhere (32, 33), the ethics committee of Basel and Northwestern Switzerland (EKNZ; Basel, Switzerland) and the ethics committee of St. Gallen (EKOS; St. Gallen, Switzerland) approved the study protocol and its amendments (EKNZ; 2016-1347). The study was carried out in accordance with the ethical principles laid down in the Declaration of Helsinki and its later amendments (43). The study was performed between March 2016 and March 2019.

### Participants

The initial sample consisted of 46 PwMS, who were treated for their MS at the center for neurorehabilitation in Valens (Kliniken Valens, SG, Switzerland). Of those, 26 (56.5%) also underwent objective sleep-EEG assessments. These 26 PwMS were approached again, and 16 (61.5%) agreed to participate at the follow-up assessment. Preliminary calculations showed that compared to non-participants of the follow-up study (*n* = 10; 9 females and 1 male), participants of the follow-up study (*n* = 16; 11 females; 5 males) did not differ as regards sex distribution, age, depression, fatigue, insomnia scores, physical activity, objective sleep efficiency, and EDSS (Expanded Disability Status Scale) score (see Table 1).

**Table 1:** Descriptive and inferential statistical comparison of sociodemographic, MS-related, psychological and sleep-related comparisons between participants, who did and did not participate at the follow-up study.

	**Groups**		**Statistics**
	**Participants**	**Non-participants**	
*N*	16	10	
*n*	*n* (%)	*n* (%)	*X*^2^-test
Gender (male/female)	5/11	1/9	*X*^2^(*N* = 26, df =1) = 2.43
	M (SD)	M (SD)	*t*-test
Age (years)	49.13 (9.61)	48.92 (9.94)	*t*(24) = 0.06
Beck Depression Inventory	1.47 (1.68)	1.08 (1.0)	*t*(24) = 0.60
Fatigue	37.14 (14.94)	34.85 (14.21)	*t*(24) = 0.41
Insomnia Severity Index	12.83 (4.60)	14.77 (4.89)	*t*(24) = 1.13
Physical activity			
Moderate physical activity (min)	827.00 (439.71)	568.75 (220.56)	*t*(24) = 0.65
Vigorous physical activity (min)	148.00 (68.92)	112.50 (56.90)	*t*(24) = 0.88
Sleep-EEG			
Sleep efficiency (%)	81.30 (11.44)	71.28 (15.29)	*t*(24) = 2.00
	Median (range)	Median (range)	*U*-test
EDSS	5 (3.00–6.00)	5 (3–6.50)	*Z* = −0.49

*EDSS, Expanded Disability Status Scale*.

### Assessments

#### Sociodemographic and MS-Related Information

As outlined elsewhere (32, 33), participants reported on their age and gender. Furthermore, EDSS scores at baseline were taken from medical records, while for EDSS scores at follow-up, participants were instructed to ask their neurologists about their current EDSS score. Next, they reported their current medications, that is, all current disease-modifying medications to treat MS, and psychopharmacological medications to treat psychiatric symptoms such as depression, sleep disturbances, and chronic pain.

#### Time Lapse Between the Baseline and Follow-Up Assessment

Based on medical records and participants' information, we calculated how many months passed between the discharge from the rehabilitation center (baseline) and the follow-up.

### Objective Sleep

As in previous studies (32, 33, 44, 45), we employed a one-channel, portable sleep-EEG recording device (Fp2-A1; electromyogram; electrooculogram; Somnowatch® Somnomedics; Randersacker, Germany). Two experienced and independent sleep lab experts visually analyzed the EEG signals according to standard procedures (46). Sleep continuity parameters consisted of (a) sleep onset latency (min), (b) total sleep time (min), and (c) duration of awakenings after sleep onset (min). Sleep architecture parameters were (a) non-REM sleep, (b) light sleep (stages 1 and 2): absolute (min) and relative duration (%), (c) slow-wave sleep/deep sleep (stages 3 and 4): absolute (min) and relative duration (%), and (d) rapid eye movement sleep (REM): absolute (min) and relative duration (%). All measurements were reported in means (M) and standard deviations (SD).

#### Self-Reported Physical Activity

As described in more details elsewhere (32, 33), participants completed the International Physical Activity Questionnaire Short Form (IPAQ-SF) to assess subjective physical activity (47). As outlined elsewhere (48), participants reported how many days per week they performed vigorous physical activity; “vigorous” was operationalized with the following: “After vigorous physical activity, I'm sweating, breathing a lot, and I feel tired”. The response categories ranged from 0 to 7 days. In addition, participants were asked to indicate the average duration (per day) for the days they engaged in these activities. Multiplication of frequency and duration scores resulted in an estimate of weekly hours invested in vigorous physical activity. To assess moderate physical activity, the items and the calculations were identical, though “moderate” was operationalized with the following: “After moderate physical activity, I feel a bit tired, though, I can breathe normally, and I do not feel tired.” Scores are always reported in M and SD.

### Statistical Analysis

To compare age between baseline and follow-up, a *t*-test for related samples was performed.

To compare dimensions of objective and subjective sleep, physical activity levels, and psychological dimensions between completers and non-completers of the study, a series of *t*-tests was performed.

To compare baseline and follow-up scores of subjective and objective sleep, depression, fatigue, and physical activity levels, a series of ANOVAs for repeated measures was performed, controlling for the time lapse between baseline and follow-up assessment (M = 13.5 months; SD = 6.00). For want of alternatives, a *t*-test for related samples was also performed for EDSS scores, always controlling for the time lapse between the assessments.

The nominal level of significance was set at alpha = 0.05. All computations were performed with SPSS® 25.0 (IBM Corporation, Armonk NY, USA) for Apple Mac®.

## Results

### Sociodemographic and Illness-Related Information

Table 2 reports all descriptive and inferential statistical indices regarding the sociodemographic and illness-related information.

**Table 2:** Descriptive and inferential statistical comparison of sociodemographic, MS-related, psychological and sleep-related comparisons between values at baseline and follow-up, always controlling for the time lapse (*M* = 13.5 months; *SD* = 6.00) between the two time points.

	**Groups**		**Statistics**	
	**Baseline**	**Follow-up**		
*N*	16	16		
*n*	*n* (%)	*n* (%)	*X*^2^-test	
Gender (male/female)	5/11	5/11	-	
	M (SD)	M (SD)	*F*-test	$\eta_{\text{p}}^{\text{b}}$
Age (years)^a^	49.12 (9.61)	50.25 (9.59)	*t*(15) = 9.00^***^	-
Beck depression inventory FS	1.47 (1.68)	1.40 (1.05)	**F**_(1, 13)_ = 1.04	0.074 (M)
Fatigue	37.14 (14.94)	50.07 (15.98)	**F**_(1, 13)_ = 2.90	0.195 (L)
Insomnia severity index	12.83 (4.60)	15.07 (6.71)	**F**_(1, 13)_ = 2.30	0.150 (L)
Physical activity				
Moderate physical activity (min/week)	827.00 (439.71)	320.00 (120.56)	**F**_(1, 13)_ = 0.17	0.013 (T)
Vigorous physical activity (min/week)	148.00 (68.92)	104.00 (59.58)	**F**_(1, 13)_ = 0.13	0.010 (T)
	M (SD)	M (SD)	*F*-test^2^	
Sleep-EEG dimensions				
Total sleep time (min)	391.00 (87.50)	417.44 (62.68)	**F**_(1, 13)_ = 0.45	0.034 (S)
Sleep efficiency (%)	82.73 (12.41)	81.06 (9.67)	**F**_(1, 13)_ = 1.12	0.074 (M)
Sleep onset latency (min)	10.78 (6.36)	15.51 (10.40)	**F**_(1, 13)_ = 0.61	0.042 (S)
WASO (time, min)	81.13 (61.30)	42.24 (4.03)	**F**_(1, 13)_ = 4.90^*^	0.259 (L)
REM sleep time (min)	86.13 (47.97)	23.07 (4.02)	**F**_(1, 13)_ = 1.36	0.088 (M)
REM (%)	20.69 (10.00)	5.13 (0.99)	**F**_(1, 13)_ = 2.02	0.126 (M)
Light sleep duration (min)	231.82 (59.55)	276.13 (45.50)	**F**_(1, 13)_ = 3.71^+^	0.209 (L)
Light sleep percentage (%)	61.39 (20.74)	58.00 (5.65)	**F**_(1, 13)_ = 0.02	0.002 (T)
Deep sleep duration (min)	83.31 (104.83)	87.97 (23.26)	**F**_(1, 13)_ = 0.11	0.008 (T)
Deep sleep percentage (%)	17.91 (9.11)	19.43 (4.86)	**F**_(1, 13)_ = 0.16	0.012 (T)
EDSS^c^	5.22 (1.27)	5.28 (1.19)	**F**_(1, 13)_ = 0.22	0.002 (T)

a *Comparison of age between baseline and follow-up was not controlled for time lapse*.

b *Always controlling for the time lapse*.

c *To compare EDSS scores, and controlling for the time lapse, a repeated ANOVA was performed*.

*FS, fast screener; EDSS, Expanded Disability Status Scale; T, trivial effect size; S, small effect size; M, medium effect size; L, large effect size*.

* *p < 0.05*;

+ * p < 0.1*;

*** *p < 0.001*.

On average, follow-up assessment was performed 13.5 months (SD = 6.00) after discharge from the rehabilitation center. Five male and 11 female PwMS participated at the follow-up assessment.

By nature, mean age increased, while the median EDSS score remained unchanged. Descriptively, fatigue increased.

Symptoms of depression and fatigue did not statistically change over time. Descriptively, however, fatigue scores increased by 34.8%.

### Subjective and Objective Sleep Dimensions

Table 2 also reports the subjective and objective sleep parameters at baseline and follow-up.

Descriptively, subjective insomnia scores increased.

As regards objective sleep measurements, wake time after sleep onset statistically significantly decreased, but the duration of light sleep increased by trend. Descriptively, REM-sleep decreased (duration and %). For all other dimensions (total sleep time; sleep onset latency; light sleep: %; deep sleep: % and min), no statistically or descriptively significant mean differences were observed.

### Self-Reported Physical Activity Levels

Table 2 also reports the moderate and vigorous physical activity levels at baseline and follow-up. Self-reported physical activity levels did not statistically decrease. Descriptively, however, moderate physical activity levels decreased for 61%, and vigorous physical activity levels decreased for 29.7%.

## Discussion

The key findings of this small longitudinal study among persons with multiple sclerosis (PwMS) and performed under naturalistic conditions were that about 13.5 months after discharge from a rehabilitation center, descriptively, symptoms of depression remained fairly stable; descriptively, fatigue and subjective sleep scores worsened; and objectively, measured wake time after sleep onset statistically significantly improved. Further, descriptively, moderate and vigorous physical activity levels decreased. The present data add to the current literature in that we did not only replicate previous works but also expanded on them: Stability and changes as regards psychological, illness-related, physical activity-related and subjective and objective sleep-related dimensions did evolve in a differentiated fashion.

Two hypotheses were formulated and these are considered in turn.

With the first hypothesis, we assumed that also objective sleep patterns remained unchanged over a time lapse of on average 13.5 months, and the general pattern confirmed this assumption and thus mirrored previous findings (37). However, we expand upon previous data in that the follow-up objective sleep assessment occurred under naturalistic and familiar conditions. Further, wake time after sleep onset statistically significantly decreased. Also, this result demands particular attention: While the quality of the data does not allow a deeper understanding of the underlying psychophysiological mechanisms, it appears that at baseline, some participants had a particularly long wake time after sleep onset (see Table 2), while others did not. This huge range of wake time duration might be astonishing, given that baseline values were assessed under laboratory-like conditions at the rehabilitation center. In contrast, we also note that at follow-up, light sleep duration significantly increased. Combining both the decreased wake-time duration after sleep onset and the longer light sleep duration, one might speculate that the longer light sleep duration increased at the expense of a shorter wake time after sleep onset. Further, this kind of “shift” had an influence neither on sleep efficiency nor on the subjectively perceived sleep quality.

With the second hypothesis, we assumed that physical activity indices remained unchanged over a time lapse of on average 13.5 months, though the answer is not that evident and we could not fully confirm previous findings (37). While from a pure statistical point of view (significant *p*-values; effect sizes), no change was observed; descriptively, both moderate and vigorous physical activity levels decreased. The explanation of this pattern of results demands more attention. First, as shown in Table 2, standard deviations were large at both time points, suggesting thus that some participants were particularly physically active, while others were not. Given this, the zero result might be the sum of particularly high and particularly low physical activity levels. Second, in the present study, baseline values equaled to the end assessment of a 3- to 4-week lasting stay at the rehabilitation center. As described elsewhere (33), the stay at the rehabilitation center consisted of regular physiotherapy and physical activity interventions. Given this, it appears plausible that both moderate and vigorous physical activity levels under guided and monitored conditions were higher, compared to an in-home and self-paced context.

The novelty of the results should be balanced against the following limitations: First, the sample size was small, though we also focused on effect size calculations, which by definition are not sensitive to sample sizes. Second, given the voluntary character of the study, only participants able and willing to comply with the study conditions took part in the study; it follows that the entire pattern of results might be biased. In this view, third, it is also conceivable that further factors such as medication intake, social structures, employment, or stable daytime structures could have biased the present pattern of results. Likewise, fourth, current change of MS status could have unfavorably interfered with dimensions of the follow-up assessment; while a current attack was not explicitly an exclusion criterium, change in MS status and diseased progress occur rather slowly (3), and participation at the study under the condition of an attack appears rather unlikely. Fifth, the female:male ratio was 11:5; given the small sample size, statistical calculations to take gender as further independent factor were not taken into consideration. It follows that a systematic gender bias could not be ruled out.

## Conclusions

The pattern of results of the present small study conducted under naturalistic conditions suggests that during a time lapse of about 13.5 months, objective sleep patterns, physical activity levels, and psychological well-being might change at an individual and fine-grained level. We hold that the present results are of clinical and practical importance for treatment counseling: PwMS can be reassured that their sleep quality does not deteriorate, once they have left a rehabilitation center. Further, they should be encouraged to keeping their physical activity levels as stable and as high as possible.

## Data Availability Statement

The raw data supporting the conclusions of this article will be made available by the authors to experts in the field upon request.

## Ethics Statement

The studies involving human participants were reviewed and approved by Ethikkommission Nordwest und Zentralschweiz (EKNZ, Basel, Switzerland): 2016-1347. The patients/participants provided their written informed consent to participate in this study.

## Author Contributions

DSB, RG, JK, JB, DC, OR, DN, MG, and SB: conceptualization. DSB, RM, JK, OR, DN, and MG: data curation. DSB, RG, JK, JB, RM, DC, OR, DN, MG, and SB: investigation and methodology. DSB, RG, JB, OR, DC, MG, and SB: project administration. DSB, RG, OR, MG, and SB: supervision. DSB, RG, JK, RM, JB, OR, DN, DC, MG, and SB: writing the draft and final version. All authors read and agreed to the publication in the present form. All authors have read and agreed to the published version of the manuscript.

## Conflict of Interest

The authors declare that the research was conducted in the absence of any commercial or financial relationships that could be construed as a potential conflict of interest.
